# Targeting cobra venom cytotoxin: a linear 40-mer ssDNA aptamer-based antivenom confers neutralisation potentials against cobra venom-induced cytotoxicity

**DOI:** 10.1007/s00204-025-04211-z

**Published:** 2025-09-30

**Authors:** Jia Jin Hiu, Hock Siew Tan, Michelle Khai Khun Yap

**Affiliations:** School of Science, Monash University Malaysia, Bandar Sunway, Jalan Lagoon Selatan, 47500 Bandar Sunway, Selangor Malaysia

**Keywords:** Cytotoxicity, Aptamer, 40T, Neutralisation, *Naja* venom

## Abstract

Cytotoxin (CTX) is one of the major cobra venom components that contributes to dermonecrosis due to its cytotoxicity. However, current antibody-based antivenoms exert limited neutralisation effects against CTX-induced dermonecrosis. This study focused on discovering aptamer-based antivenom that specifically targets CTX, using repetitive centrifugation-based Systematic Evolution of Ligands by EXponential enrichment (SELEX) selection approach and Illumina amplicon next-generation sequencing. A total of 12 repetitive centrifugation-based selection rounds including a negative selection between rounds 7 and 8 were performed. This was followed by amplicon next-generation sequencing and sequencing bioinformatics workflow to analyse the abundance and persistence of the CTX-binding candidates. Sequences with log_10_ read counts of 2–3 with a round representation of 3–4 were selected as the final candidates. A linear and single-stranded DNA, 40T, was discovered and it exhibited high binding affinity and specificity to CTX with dissociation constant (*K*_D_) of 0.33–0.41 µM, as demonstrated by direct and competitive enzyme-linked aptamer assay (ELAA). 40T acquired a ‘sandwich’ configuration binding to CTX at the functional epitopes. It exhibited neutralisation potency against the CTX-induced cytotoxicity with EC_50_ of 0.47 µM. To mimic the real envenomation situation, venoms from *Naja sputatrix*, *Naja siamensis,* and *Naja sumatrana* were used to induce experimentally envenomed model for treatment with 40T. 40T demonstrated notable cell viability-restoring effects against these venoms at low micromolar ratios. These findings suggested a modified selection and sequencing workflow to discover the potential of 40T as aptamer-based antivenom to mitigate venom-induced dermonecrosis.

**Supplementary Information:**

The online version contains supplementary material available at 10.1007/s00204-025-04211-z.

## Introduction

Snakebite envenoming (SBE) contributes to significant global mortality every year and approximately 400,000 cases of permanent morbidity (Gutiérrez et al. 2017). This neglected tropical disease is highly observed in South Asia and sub-Saharan Africa, as they encounter socioeconomic vulnerability and diminished healthcare resources (Roberts et al. 2022). Cobra (*Naja* sp.) is listed as category 1 highest medically important species by the World Health Organization. Dermonecrosis, also known as local tissue damage, is a common clinical symptom of cobra envenomation. It is often associated with tissue swelling, blistering, and tissue necrosis. In severe cases, victims might suffer permanent disability from limb amputation and secondary infections. Dermonecrosis is highly attributed to substantial levels (40–70%) of cytotoxin (CTX) found in cobra venoms. CTX is a basic polypeptide consisting of 59–62 amino acids, with a molecular weight of approximately 7 kDa (Munawar et al. 2018). It has a three-finger loop structure flanked by five antiparallel β sheets and a central hydrophobic region (Gasanov et al. 2014). The three loops of CTX are flanked by basic lysine and arginine residues, which are responsible for cell membrane interactions (Dubovskii and Utkin 2014) resulting in direct cell lysis. In addition, CTX has also been found to activate different apoptosis-related signalling pathways (Hiu and Yap 2021; Lin et al. 2010; Teoh and Yap 2020; Zhang and Cui 2007), mitochondrial membrane depolarisation (Gasanov et al. 2014; Teoh and Yap 2020), lysosomal damage (Wu et al. 2013), necrosis (Ebrahim et al. 2015), and necroptosis (Hiu and Yap 2021). The inconclusive cytotoxic mechanisms accentuate uncertainties for the underlying causes of dermonecrosis and causing treatment for dermoncrosis more challenging.

It is well known that the three loops of CTX are the functional sites and epitopes of CTX (Hiu et al. 2023). Thus, its functional epitope loops can serve as enriched targets (Hiu et al. 2023) for new pathology-specific antivenoms (Ahmadi et al. 2020; Alomran et al. 2022; Knudsen and Laustsen 2018). The current available and effective treatment for cobra envenomation is antibody-based antivenoms, which are manufactured from the purification of immunoglobulins from hyperimmunised animals, resulting in high production costs. Hypersensitivity reactions have also been reported after antivenom administration. More importantly, the antivenoms are ineffective against dermonecrosis due to the low immunogenicity of CTX, which is attributed to its three-finger folded structure, limiting its surface exposure to elicit an immune response (Chanda et al. 2019; Chanda and Mukherjee. 2020; Kalita et al. 2018; Liu et al. 2020; Patra and Mukherjee 2020). Its three-finger folded structure also restricts its surface exposure to elicit an immune response (Liu et al. 2020).

Aptamers are strong potential candidates used as alternative neutralising molecules. They are short, single-stranded oligonucleotides discovered through multiple selection cycles, known as Systematic Evolution of Ligands by Exponential Enrichment (SELEX), which can select target-binding aptamers with high binding affinity and specificity (Ascoët and De Waard 2020). Studies have shown that aptamers offer superior advantages because of their better thermal stability and low immunogenicity (Aljohani et al. 2022; Dhar et al. 2020; Keefe et al. 2010; Song et al. 2012). In addition, aptamers can be easily modified for optimisation or conjugation with secondary reagents depending on their therapeutic purpose (Li et al. 2023). Different examples of aptamers have been discovered against kraits (*Bungarus* sp.) venom toxins, for example, a DNA aptamer that targets α-bungarotoxin from *B. multicinctus* and has a high binding affinity of 7.58 µM (Lauridsen et al. 2012). In addition, by using the plate-SELEX method, aptamers against β-bungarotoxin from *B. multicinctus* venom with nanomolar-scale binding have also been discovered (Ye et al. 2014). This type of cross-reactivity was also observed in aptamers selected against the SVSP from *Calloselasma rhodostoma* venom and *batroxobin* from *Bothrops atrox* venom, with their ability to inhibit fibrinogenolysis (Alomran et al. 2022). Besides snake venoms, aptamers have also been found to effectively neutralise other animal venom toxins such as conotoxins from cone snail venoms: for example, DNA aptamers with high efficacy for in vitro and in vivo neutralisation for αC-conotoxin PrXA with interference to the binding of toxin for its receptor (El-Aziz et al. 2017; Taiwe et al. 2019).

Recently, we have discovered tRNA-mimetic aptamer against CTX using computational approach based on specific genomic tRNA database (Hiu et al. 2024). Nevertheless, the computational method highly depends on existing database which could restrict the initial selection library and novelty in the aptamer sequences. In this study, we attempted a modified SELEX approach, known as repetitive centrifugation-based selection based on a random oligonucleotide library. This was followed by amplicon next-generation sequencing coupled with bioinformatics workflows to maximise the CTX-specific aptamers library. The binding strength and specificity of the aptamer were determined. The neutralisation potency and efficacy of this specific aptamer were also examined against CTX and a venom-envenomed skin model to validate its efficacy as a biotherapeutic against dermonecrosis.

## Materials and methods

### Materials

Random oligonucleotide library and biotinylated aptamer were synthesised by Integrated DNA Technologies (IDT), Singapore. NHS-activated Sepharose resin and NAP-10 column was procured from Cytiva, USA. GelRed^®^ nucleic acid gel stain was obtained from Biotium, USA. 3,3′,5,5′-Tetramethylbenzidine (TMB) substrate was purchased from R&D Systems, USA. Cell-counting kit (CCK-8) assay was obtained from Dojindo, Germany. Human skin keratinocytes, HaCaT (EP-CL-0090), was purchased from Elabscience^®^, USA. Venoms from *Naja sputatrix* and *Naja siamensis* were purchased from VenomTech, UK.

### Oligonucleotide library and CTX preparation

A random and single-stranded DNA (ssDNA) oligonucleotide library of 76 nucleotides in length, comprising a central randomised region of 40-mer and 18-mer flanking sequences at both ends (5′-ATCCAGAGTGACGCAGCA-N40-TGGACACGGTGGCTTAGT-3′), was synthesised at 250 nmol (Sefah et al. 2010). The oligonucleotide library (250 nmol of yield) was then dissolved in 250 μL of nuclease-free water to obtain a final concentration of 1 μM. The reconstituted oligonucleotide library was heated for 5 min at 95 °C and cooled to room temperature for folding. A conserved CTX sequence from *Naja* species was used as the target CTX, as it represents the highly conserved CTX of different isoforms (Misuan et al. 2023).

> conserved CTX sequence

LKCNNKLVPLFYKTCPAGKNLCYKMFMVSTPTKVPVKRGCIDVCPKNSLLVKYVCCNTDRCN

### Immobilisation of CTX

In brief, 200 μL of NHS-activated Sepharose resin (Cytiva, USA) was coupled with 100 µg of CTX in coupling buffer (0.2 M NaHCO_3_, 0.5 M NaCl, pH 8.3) and blocked with 100 µL of quenching buffer (0.1 M Tris–HCl, pH 8.5). Both the coupling and quenching steps were conducted at 4 °C overnight with end-to-end mixing. The CTX-immobilised Sepharose resin was then washed twice with 150 µL of column washing buffer (0.1 M Tris–HCl, pH 8.5, 0.1 M acetate buffer, and 0.5 M NaCl, pH 4.7) alternately and equilibrated with 100 µL of aptamer binding buffer (20 mM Tris–HCl, pH 7.6; 120 mM NaCl; 5 mM KCl; 1 mM MgCl_2_; and 1 mM CaCl_2_).

### Repetitive centrifugation-based SELEX

The CTX-immobilised Sepharose was incubated with 250 µL of 30 nmol of the random oligonucleotide library at room temperature for 2 h with end-to-end mixing. Binding buffer containing 0.2% Tween-20 was added, followed by centrifugation at 14,800 rpm for 5 min to remove unbound oligonucleotides. Then, an elution buffer (1.5 M NaCl in 10 mM Tris, pH 7.2) was added to elute the bound oligonucleotides from the CTX-immobilised Sepharose through another centrifugation at 14,800 rpm for 5 min. The bound oligonucleotides were amplified via polymerase chain reaction (PCR), followed by strand separation before proceeding with the subsequent 12 selection rounds. Negative selection was included between rounds 7 and 8 to remove nonspecific aptamers.

### Amplification of CTX-bound oligonucleotides

The CTX-bound oligonucleotides were then amplified via polymerase chain reaction (PCR) via forward primers (5′-ATCCAGAGTGACGCAGCA-3′) and biotinylated reverse primers (5′-Biotin-ACTAAGCCACCGTGTCCA-3′) on the basis of the PCR conditions in Table 1. Two rounds of PCR were performed to obtain sufficient oligonucleotides for the subsequent selection rounds (Narayan et al. 2022), using the amplified pool as the template (the template volume added was 10% of the total reaction mixture; Table 2). The same forward and reverse primers were used as in the previous round of PCR. The final volume of 50 µL per reaction was adjusted with Milli-Q H_2_O.

**Table 1:** PCR condition for the first selected pool of CTX-bound oligonucleotides

	Initial denaturation	Denaturation	Annealing	Extension	Final extension	Rest
Temperature (°C)	94	94	54	72	72	4
Time (min)	00:30	00:30	00:30	01:00	05:00	∞
Cycle (s)	1	30			1	∞

**Table 2:** PCR condition for the amplified pool of CTX-bound oligonucleotides

	Initial denaturation	Denaturation	Annealing	Extension	Final extension	Rest
Temperature (°C)	94	94	54	72	72	4
Time (min)	00:30	00:30	00:30	01:00	05:00	∞
Cycle (s)	1	8			1	∞

### Strand separation of oligonucleotides from the PCR product

A streptavidin agarose column was prepared for strand separation of oligonucleotides from the biotinylated PCR product. The column was first washed three times with washing buffer (20 mM Tris–HCl, pH 7.5, and 0.15 M NaCl). The PCR-amplified oligonucleotide pool was added to the streptavidin agarose column and incubated for 15 min at room temperature with end-to-end mixing. After removing the unbound fraction, strand separation was conducted with 200 µL of 0.2 M NaOH to obtain the nonbiotinylated ssDNA, while the biotinylated strands were eluted in 200 µL of 3 M MgCl_2_. The column was washed with washing buffer three times between each step.

### Purification of oligonucleotide

The nonbiotinylated and biotinylated oligonucleotides were desalted via an NAP-10 column (Cytiva, USA) following the manufacturer’s protocol. In general, the column was washed with 15 mL of Milli-Q H_2_O prior to loading the oligonucleotides. The oligonucleotides were then eluted with 1.5 mL of Milli-Q H_2_O. The nonbiotinylated oligonucleotides were subjected to subsequent rounds of selection, while the biotinylated aptamers were PCR amplified (Table 1) for Illumina amplicon sequencing.

### Illumina amplicon sequencing

The amplicon library was prepared by attaching the Illumina Unique Dual Indices (Illumina, CA, USA) to the amplified product via PCR. The amplicon library was cleaned via AMPure beads. Amplicon libraries were analysed for concentration via Biodrop µLITE+. Libraries were normalised to 1.75 nM and pooled before loading onto the NovaSeq 6000 sequencing platform (Illumina, CA, USA). Paired-end sequencing was performed with 20 k reads per library. A quality check of the DNA libraries was performed via the FastQC tool to ensure that the sequencing was successful.

### Bioinformatics analysis

The sequencing data were analysed via the Galaxy Analyses Web Server (https://usegalaxy.org/) on the basis of an established workflow for aptamer high-throughput sequencing data (Thiel 2016). In brief, the FASTA files were sorted according to the intact 5′ constant region with the ‘Barcode Splitter’ tool. The 5′ and 3′ constant regions and adapter sequences were then removed via the ‘Trim sequences’ tool. Next, the sequences were filtered to retain variable regions with the lengths of 38–42 nucleotides, according to the method described by Thiel (2016). Although the variable region of the oligonucleotide library consisted of 40-mer random bases, we performed a ± 5% length filter to account for the probability of sequencing discrepancy. A nonredundant database (NrD) was generated by compiling the aptamer data from multiple selection rounds into a single comprehensive database. This was achieved through the ‘*Nrd Workflow 5 Rounds*’ that was available in the ‘Published Galaxy Workflows’. To compare the distribution of read counts within round 0 (oligonucleotide library) to read counts in selection rounds 1, 2, 3, and 4, the ‘*Abundance NrD Analysis Workflow’* was acquired, whereas the ‘*Persistence NrD Analysis Workflow*’ was adopted to compare the number of rounds in which the aptamer sequence appears (round representation) within round 0 to those found in selection rounds 4, 7, 8, and 11. The sequence with the most reads was selected as the final aptamer candidate.

### Molecular docking

Molecular docking analysis was performed to study the binding between the potential aptamers and CTX via the High Ambiguity Driven protein‒protein DOCKing (HADDOCK 2.4) server (https://rascar.science.uu.nl/haddock2.4/ (Honorato et al. 2024). The docking complexes were visualised via Biovia Discovery Studio 2021 (Dassault Systèmes) and subjected to RING4.0 analysis (https://ring.biocomputingup.it/) to identify the interacting residues within the complex (Del Conte et al. 2024).

### Electrophoretic mobility shift assay (EMSA)

EMSA was performed to visualise the shift in electrophoretic mobility upon the binding of an aptamer to CTX. If complex formation occurs between an aptamer and CTX, a difference in the migration rate can be observed due to the larger molecular size of the complex. In brief, 100 ng of aptamer was incubated with CTX in SELEX binding buffer following mass-to-mass ratios of 1:3, 1:5, 1:7, 1:10, and 1:15 for 1 h at 37 °C. The relative mass for CTX for each mass-to-mass ratio was 50 ng, 100 ng, 300 ng, 500 ng, and 700 ng, respectively. The complexes were then loaded onto a 0.7% agarose gel and run at 120 V for 20 min. The gel was viewed after 15 min of staining with GelRed^®^ nucleic acid gel stain (Biotium, USA). Both the aptamer-only control and the CTX control were included in this assay.

### Direct enzyme-linked aptamer assay (ELAA)

To determine the binding affinity of the aptamer, 10 ng/well CTX in carbonate coating buffer, pH 9.6, was coated onto a 96-well ELISA plate and incubated overnight at 4 °C. The wells were blocked with 1% BSA in PBS for 1 h at room temperature. The biotinylated aptamer was synthesised by Integrated DNA Technologies, IDT (Singapore), folded by heating at 95 °C, and then incubated at room temperature for 30 min. The aptamer was added and incubated with CTX at 37 °C for 1 h in SELEX binding buffer. HRP-conjugated streptavidin in 1% BSA was added at a 1:40 ratio, and the mixture was incubated at 37 °C for another hour in the dark. The plate was washed six times with 0.01 M PBS (pH 7.4) containing 0.05% Tween 20 (PBS-T) between every step. Then, 3,3′,5,5′-tetramethylbenzidine (TMB) substrate (R&D Systems, USA) solution was added to each well, and the samples were incubated for 20 min in a dark at room temperature. This reaction was stopped with 0.1 M H_2_SO_4_, and the absorbance readings were measured at 450 nm with a Tecan plate reader (Sunrise, Austria). The equilibrium dissociation constant (*K*_D_) values for the aptamers were determined via the one-site specific binding equation from GraphPad Prism 9 software, where the equilibrium dissociation constant (*K*_D_) is the concentration of the aptamers required to reach 50% of the maximal binding.

### Competitive enzyme-linked aptamer assay (ELAA)

To determine the binding specificity of the aptamer, competitive ELAA was carried out using a random oligonucleotide (5′-ATCGGATTATAATTCACCAAGTCAGATTTTTTGATCTC-3′) as the competitor. Like direct ELAA, 10 ng of CTX was coated onto a 96-well ELISA plate and incubated at 4 °C overnight. After washing six times with PBS-T, nonspecific binding sites were blocked with 1% BSA in PBS for 1 h at room temperature. After three washing steps, the aptamers at concentrations of 0.1, 1, 10, and 100 nM were incubated with CTX following the conditions described in the direct ELAA. The random oligonucleotide at 1 nM in the binding buffer was added to each well to allow binding competition. This reaction occurred at 37 °C for 1 h, followed by the addition of HRP-conjugated streptavidin (1:40). The *K*_D_ value obtained in this assay was compared with the *K*_D_ from direct ELAA to determine the binding efficacy of the aptamer in the presence of a competitor.

### In vitro neutralisation of cytotoxicity assay

Since CTX is known to induce cytotoxicity, the neutralising effect of the aptamer was examined through a cytotoxicity cell-counting kit (CCK-8) assay (Dojindo, Germany). In this study, human skin keratinocytes and HaCaT (Elabscience^®^, USA) cells were used to mimic real-world cytotoxic conditions during envenomation. HaCaT cells were seeded in a 96-well plate in serum-free DMEM at a density of 50,000 cells/mL. The aptamer was preincubated with 9 µM CTX at different aptamer: CTX molar ratios ranging from 0.03125 to 1 M for 1 h at 37 °C to form complexes before being added to HaCaT cells for 24 h of treatment. Cell viability was assessed via the CCK-8 reagent, and the absorbance readings were measured at 450 nm. The EC_50_ of aptamer was determined by GraphPad Prism 9 software, where the EC_50_ is the concentration of the aptamer that exhibits a half-maximal neutralising effect, indicating the neutralising potency of the aptamer.

### Aptamer-mediated CTX rescue in experimental post-envenomed assay

The rescue potency of the aptamers in an experimental post-envenomated cell model was determined by exposing HaCaT cells (50,000 cells/mL) to 9 µM CTX for 6 h at 37 °C. Then, the aptamers in the binding buffer were added to the envenomated cells for 24 h at different aptamer: CTX molar ratios ranging from 0.03125 to 1 M. Cell viability was determined via the CCK-8 reagent as described above. A monoclonal antibody, anti-CTX single-chain variable fragment (scFv) TPL0027_01_F7 (Ahmadi et al. 2020) was included as a positive control. The EC_50_ of aptamer was determined via GraphPad Prism 9 software, where the EC_50_ is the concentration of aptamer that can rescue 50% of HaCaT cells.

### Aptamer-mediated crude venom rescue in experimental post-envenomed assay

In this study, venoms of Category 1 medically important cobra species, namely, *Naja sputatrix* (VenomTech, UK), *Naja siamensis* (VenomTech, UK), and *Naja sumatrana* (Peninsular Malaysia), were used to induce the experimental envenomation of HaCaT cells. HaCaT cells (50,000 cells/mL) were treated with the IC_50_ of each venom for 6 h before they were treated with the aptamer at different concentrations ranging from 0.125 to 2 µM for 24 h. A rescue assay was used to assess the inhibitory effect of the aptamer on venom-induced cytotoxicity. Cell viability was determined using CCK-8 reagent, and the absorbance was measured at 450 nm.

### Statistical analyses

All the experimental data are expressed as the mean ± standard error of the mean (S.E.M.) for three biological replicates and technical replicates. Two-way ANOVA with Tukey’s multiple post hoc comparison test on GraphPad Prism 9 was used to compare the mean significance of the data within and between each experimental group. Differences in means when the *p *value was < 0.05 were considered statistically significant.

## Results and discussions

### Quantification and selection of aptamers for Illumina amplicon sequencing

**Fig. 1:**
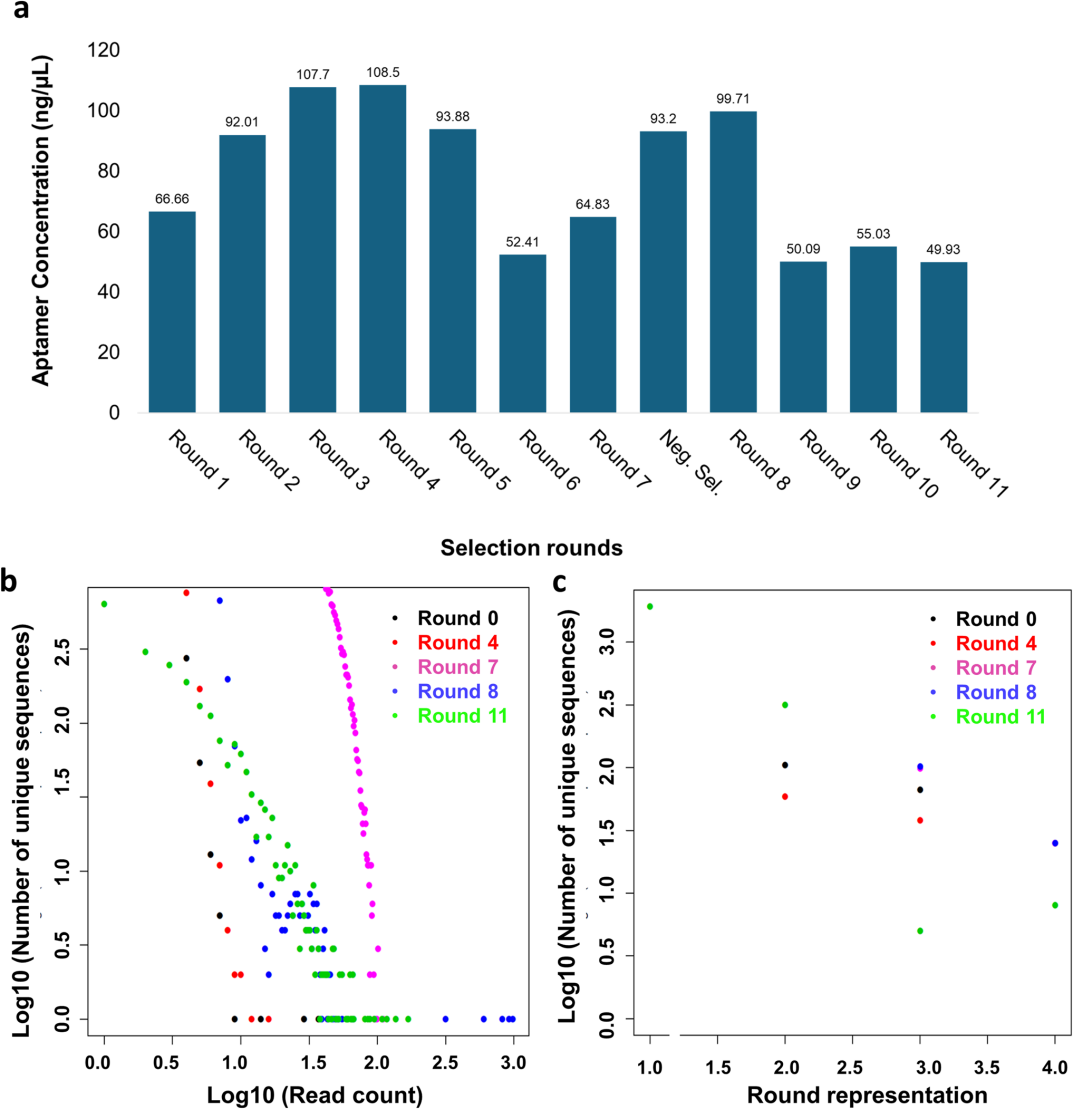
Repetitive centrifugation-based selection and amplicon sequencing discovery of CTX-specific aptamer. **a** Aptamer concentrations (ng/μL) from the 12 selection rounds. A negative selection was included between the seventh and eighth rounds to remove non-specific aptamers. Graphical illustrations of the **b** abundance and **c** persistence NrD analyses plotting the read count and round representation, respectively, of a non-selected control round 0 and four selection rounds (rounds 4, 7, 8 and 11)

Since its introduction in the early 1990s, SELEX has been the gold standard for discovering aptamers that bind specifically to targets, which involves several common steps, such as template preparation, oligonucleotide purification, and recovery of bound aptamers (Narayan et al. 2022). The current study customises this approach to a repetitive centrifugation-based approach for the selection and enrichment of aptamers that specifically target CTX. This approach is suitable for low-molecular-weight targets at a relatively smaller scale. A total of 12 selection rounds consisting of 11 positive selections and 1 negative selection were performed to obtain the final pool of CTX-specific aptamers. Like in most previous studies (Kim et al. 2013; Saad et al. 2020; Shaukat et al. 2024), each round of aptamer concentration was monitored spectrophotometrically to ensure the high purity of the aptamers, with an A260/A280 ratio of approximately 1.8. Measuring the aptamer concentration provides insights of whether enrichment and saturation of selection have been achieved. The selection enrichment indicates the accumulation of aptamers with increasing binding affinity to the target CTX over successive selection rounds. When most of the aptamers are the high-affinity binders for CTX, successive selection rounds will result in minimal further enrichment. Therefore, an increase in concentration after each selection suggests an enrichment of high-affinity and specific binding aptamers for CTX, as compared to the initial aptamer pool. When a saturation of selection is achieved, then the concentrations of aptamers remain consistent. As shown in Fig. 1a**,** the aptamer concentration started to decrease and reached a plateau between rounds 9 and 11, indicating that saturated selection was achieved. The aptamers from rounds 0, 4, 7, 8, and 11 were chosen for amplicon next-generation sequencing because round 0 was the original aptamer library; round 4 had the highest aptamer concentration (ng/μL); rounds 7 and 8 were the pre- and post-negative selection rounds, respectively; and round 11 was the final selection round.

the amplicon sequencing data were processed via the galaxy analyses web server (https://usegalaxy.org/) according to an established workflow for aptamer high-throughput sequencing data (Thiel 2016). The processed amplicon sequencing data across the SELEX selection rounds 0, 4, 7, 8, and 11 are listed in Table 3, which summarises the diversity and convergence of aptamer sequences during the selection. As shown in Table 3, the aptamers were enriched with minimal selection during the first four rounds, as the aptamer concentration increased, but the complexity was similar to that of round 0, which was the original aptamer library. The round 4, with the highest aptamer concentrations (Fig. 1a), represents the slight enrichment with moderate increase of unique sequences compared to round 0. The selection of CTX-specific aptamers subsequently began from rounds 5–7, resulting in a decrease in both the aptamer concentration and the number of unique sequences. The number of unique sequences increased at round 7 with a decline of aptamer concentrations, suggesting an increased selection of CTX-specific aptamers. This was due to the removal of unbound aptamers, and left with the specifically bound aptamers, leading to fewer and less diverse aptamers.

**Table 3:** Processed amplicon sequencing data

	Number of reads				
	Round 0	Round 4	Round 7	Round 8	Round 11
Raw sequences	142592	233315	4492139	5622628	4587913
Clipped sequences	141819	232578	4480666	5592201	4584165
Trimmed sequences	141813	232462	4459013	5590262	4578564
Filtered sequences by length (min: 38; max: 42)	140777	212690	3342681	5464787	577513
No. of unique sequences	122368 (86.92%)	175341 (82.44%)	706654 (21.14%)	4162734 (76.17%)	210140 (36.39%)

After the negative selection round (between rounds 7 and 8), nonspecific-binding aptamers were eliminated, leaving a highly enriched pool of specific aptamers. This was reflected in the increased aptamer concentration and the number of unique sequences from rounds 7 to 8. Further selection rounds increased the stringency of the aptamer binding affinity and specificity, which caused a reduction in the aptamer concentration and number of unique sequences. As observed in the final selection round (round 11), there was further reduction of sequence diversity, indicating a convergence toward a few dominant and high-affinity sequences.

### Nonredundant database (NrD) analysis: abundance and persistence assessment of aptamers

Using the same established workflow of aptamer high-throughput sequencing data (Saad et al. 2020), a nonredundant database (NrD) was generated by compiling the unique sequences from selection rounds 0, 4, 7, 8, and 11. The abundance and persistence of each unique sequence were further analysed to monitor their read counts and round representations. The read count was defined as the total number of a specific aptamer sequence in that particular selection round, whereas round representation refers to the frequency with which an aptamer sequence was presented (Thiel 2016). An ideal selection should include an aptamer pool with increased abundance and constant persistence throughout the selection. Aptamers with these features will be filtered and selected as potential CTX-targeting candidates.

The abundance analysis (Fig. 1b) indicated that sequences in round 0 (black dots) had a log_10_ read count of 1. In other words, the subsequent selection rounds identified fewer than ten aptamer sequences from the original library. In comparison, the pool obtained at round 11 (green dots) predominantly consisted of sequences with log_10_ read counts of approximately 2.5 reads. The results suggested that sequences with log_10_ read counts of more than 2 were CTX-targeting aptamers.

The persistence analysis (Fig. 1c) indicated that approximately 100 sequences from round 0 (black dots) had a round representation of 2–3. These results suggested that approximately 100 sequences within round 0 were observed in fewer than three selection rounds. On the other hand, round 11 (green dots) contained aptamer sequences with round representations of more than 3. Therefore, sequences with more than three round representations were the desired aptamer candidates.

Alternatively, the log_10_ read count (Fig. 1b) and round representation (Fig. 1c) after the negative selection round (> round 8) were also considered when selecting the filtering parameter. The aptamer sequences at round 8 presented a log_10_ read count of more than 2 and a round representation of more than 3. Taken together, these criteria of abundance (> 2 log_10_ read counts) and persistence (> 3 round representations) were used as the parameters to filter the aptamer sequences. The filtered outputs are summarised in Table 4. There were three sequences with the highest sum of read counts of 1054, 1004, and 919, which represented 38-mer, 39-mer, and 40-mer ssDNAs, respectively. The binding of these ssDNAs with CTX was predicted by HADDOCK 2.4. We used HADDOCK score to rank the docking complexes, as it is the statistical parameter whereby a lower value indicates a more energetically stable complex (Ramirez et al. 2020; van Zundert et al. 2016). From the results displayed (Table 5), 40-mer ssDNA had the lowest HADDOCK score value of − 14.0 ± 16.3, suggesting energetically stable interaction with CTX. This 40-mer ssDNA was named as 40T and thus was selected for subsequent validation studies. Nevertheless, all the 38-mer, 39-mer, and 40-mer ssDNAs contained repetitive thymine (T) bases. Notably, these sequencing data consisting of numerous repetitive T bases are known to be associated with sequencing difficulties (Treangen and Salzberg 2011). This could result in a shorter read output and a drastic reduction in the number of sequences when filtered by length (38–42 bases; Table 4).

**Table 4:** Top 25 reads of the filtered aptamer sequences from rounds 0, 4, 7, 8, and 11

Sequences	Round representation	Read counts from the selection rounds					Sum of read counts
		0	4	7	8	11	
5′-TTTTTTTTTTTTTTTTTTTTTTTTTTTTTTTTTTTTTTTTTT-3′	4	9	10	27	315	3	364
5′-TTTTCTTTTTTTTTTTTTTTTTTTTTTTTTTTTTTTTTT-3′	4	1	1	1	6	0	9
5′-TTTTTTTTTTTTTTTTTTTTTTATTTTTTTTTTTTTTT-3′	4	2	2	1	22	0	27
5′-TTTTTTTTTTTTTTTTTTTTTTTATTTTTTTTTTTTTTTT-3′	4	4	1	3	36	0	44
5′-TTTTTTTTTTTTTTATTTTTTTTTTTTTTTTTTTTTTTTT-3′	4	2	1	11	32	0	46
5′-TTTTTTTATTTTTTTTTTTTTTTTTTTTTTTTTTTTTTT-3′	4	3	1	4	24	0	32
5′-TTTTTTTTTTTTTTTTTTTTTATTTTTTTTTTTTTTTTTT-3′	4	5	1	1	30	0	37
5′-TTTTTTTTTTTTTTTTTTTTTTATTTTTTTTTTTTTTTTT-3′	4	1	1	3	22	0	27
5′-TTTTTTTTTTTTTTTTTTTTATTTTTTTTTTTTTTTTTT-3′	4	1	1	4	35	1	42
5′-TTATTTTTTTTTTTTTTTTTTTTTTTTTTTTTTTTTTTTT-3′	4	1	1	3	36	0	41
5′-TTTTTTTTTTTTTTTTTTTTTTTTTTTTTTATTTTTTTTT-3′	4	3	1	2	16	0	22
5′-TTTTTTTTTTTATTTTTTTTTTTTTTTTTTTTTTTTTTT-3′	4	1	1	2	35	0	39
5′-TTTTTTTTTTTTTTTTTTTTTTTTTTTTTTTTTTTTTTTT-3′	4	37	5	56	819	2	919
5′-TTTTTTTTTTTTTATTTTTTTTTTTTTTTTTTTTTTTTTT-3′	4	4	1	1	45	1	52
5′-TTTTTATTTTTTTTTTTTTTTTTTTTTTTTTTTTTTTTTT-3′	4	3	2	2	26	1	34
5′-TTTTTTTTTTTTTTTTTTTTTTTTTTTATTTTTTTTTTTT-3′	4	3	2	1	22	0	28
5′-TTTTTTTTTTTTTTTTTTTTTTTTTTTTTTTTTTTTTTT-3′	4	14	12	55	919	4	1004
5′-TTTATTTTTTTTTTTTTTTTTTTTTTTTTTTTTTTTTTT-3′	4	2	1	3	47	0	53
5′-TTTTTTTATTTTTTTTTTTTTTATTTTTTTTTTTTTTTTT-3′	4	1	1	2	1	0	5
5′-TTTTTTTTTTTTTTTTTTTTTTTTTTTTTTTTTTTTTTTTT-3′	4	29	16	34	600	5	684
5′-TTTTTTTTTTTTTTTTTTTTATTTTTTTTTTTTTTTTTTT-3′	4	5	1	5	29	0	40
5′-TTTTTTTTTTTTTTTTTTTTTTTTTTTTTTTTTTTTTT-3′	4	5	9	55	978	7	1054
5′-TTTTTTTTTTTTTTTTTTTATTTTTTTTTTTTTTTTTTTT-3′	4	3	1	6	34	0	44
5′-TATTTTTTTTTTTTTTTTTTTTTTTTTTTTTTTTTTTTT-3′	4	1	2	2	41	0	46
5′-TTTTTTTTTTTTTTTTTTTTTTTTTTTTTATTTTTTTTTT-3′	4	1	1	3	26	0	31

**Table 5:** HADDOCK analysis of the 38-mer, 39-mer, and 40-mer ssDNA with CTX

Parameters	38-mer	39-mer	40-mer
Cluster size	3	1	5
HADDOCK score	11.7 ± 11.0	27.6 ± 7.4	− 14.0 ± 16.3
*Z*-score	− 1.9	− 1.3	− 1.6
RMSD	17.3 ± 0.3	19.3 ± 0.3	14.7 ± 0.2
*Van der Waals* energy	− 75.6 ± 3.6	− 55.7 ± 2.4	− 71.3 ± 2.1
Electrostatic energy	− 99.3 ± 20.3	− 123.8 ± 12.4	− 145.6 ± 34.5
Desolvation energy	18.7 ± 3.5	13.3 ± 3.1	13.0 ± 3.7
Restraint’s violation energy	884.1 ± 80.3	947.7 ± 80.2	735.0 ± 119.5
Buried surface area	1790.4 ± 130.1	1477.9 ± 40.2	1923.7 ± 39.6

### Binding analysis of 40T: structure, binding affinity, and specificity

**Fig. 2:**
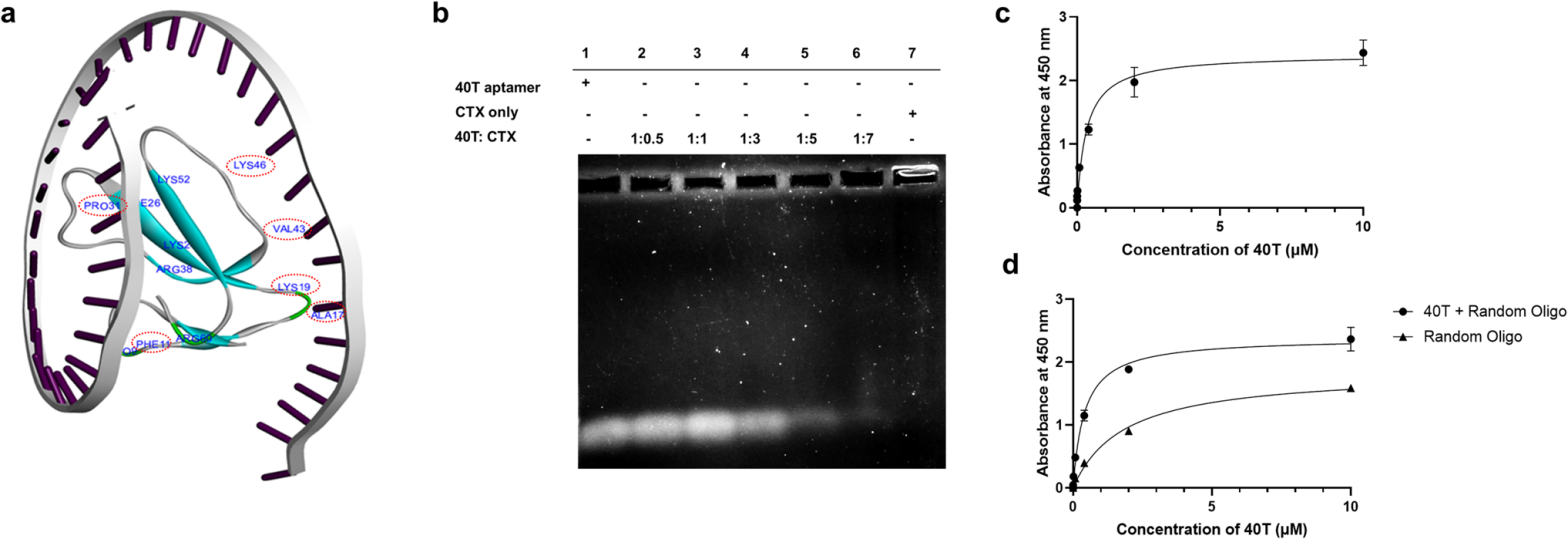
**a** Structure of 40T-CTX visualised using Biovia Discovery Studio. The red circles annotated the functional loops’ residues of CTX interacting with 40T. **b** Detection of complex formation between 40T and CTX through electrophoretic mobility shift assay (EMSA). Different 40T:CTX (mass:mass) ratios were subjected to a 0.7% agarose gel and electrophoresis at 120 V for 20 min. **c** Direct ELAA between 40T and CTX. CTX (0.1 µg/mL) was incubated with increasing concentrations of aptamer (0.00064–10 µM). **d** Competitive ELAA of 40T at different concentrations (0.00064–10 µM) in the presence of 0.08 µM of random oligonucleotide of similar length as a competitor. Data points represent the mean ± S.E.M. of biological and technical replicates (*n* = 3). The data were plotted into non-linear regression curves using GraphPad Prism 9 software. The dissociation constant (*K*_D_) of 40T in the direct and competitive ELAA was 0.33 µM and 0.41 µM, respectively

Unlike most previous studies (Bing et al. 2010; DeRosa et al. 2023; Luo et al. 2010; Roxo et al. 2019; Saccà et al. 2005), functional aptamers require folding into their structural motifs, such as G-quadruplexes and stem loops, 40T possesses an overall linear structure. Despite its linearity, 40T was observed to configure a ‘sandwich’ structure surrounding CTX (Fig. 2a). This binding conformation allows 40T to have increased contact area with the functional loops’ residues within CTX.

Additionally, the binding of 40T to CTX was further validated by EMSA and ELAA. The EMSA visualises the binding pattern of the 40T-protein complex through a migration shift due to the larger molecular size of the 40T-protein complex. This was evidenced by a decreasing band intensity from the EMSA at the bottom of the agarose gel (Fig. 2b). With increasing concentrations of CTX, more 40T was involved in aptamer‒protein complex formation. Therefore, fewer free aptamers migrated within the agarose gel. Moreover, this concentration-dependent binding of 40T to CTX was also reflected in the direct ELAA, which initially increased linearly from 0.00064 to 0.4 µM and then started to plateau towards 10 µM (Fig. 2c). The *K*_D_ value determined from direct ELAA was 0.33 µM. This finding was also consistent with a study in which α-bungarotoxin aptamers which showed neutralising effects against *N. atra* venom CTX3 had similar *K*_D_ values (0.26–2.25 µM) (Chen et al. 2016). In addition, their aptamers were also approximately 40 bases long, suggesting the optimal length of CTX-targeting aptamers (Chen et al. 2016).

The binding specificity of 40T was also confirmed through competitive ELAA, which was performed by including a random oligonucleotide with a similar sequence length to compete for the binding of CTX. In the presence of the competitor, the binding between 40T and CTX showed a similar trend as that of direct ELAA, but with a slightly higher *K*_D_ value of 0.41 µM (Fig. 2d). This result indicated that 40T was specific to CTX, as suggested by the comparable *K*_D_ values between the presence and absence of a competitor.

### In vitro neutralisation and experimental post-envenomed rescue efficacy of 40T

**Fig. 3:**
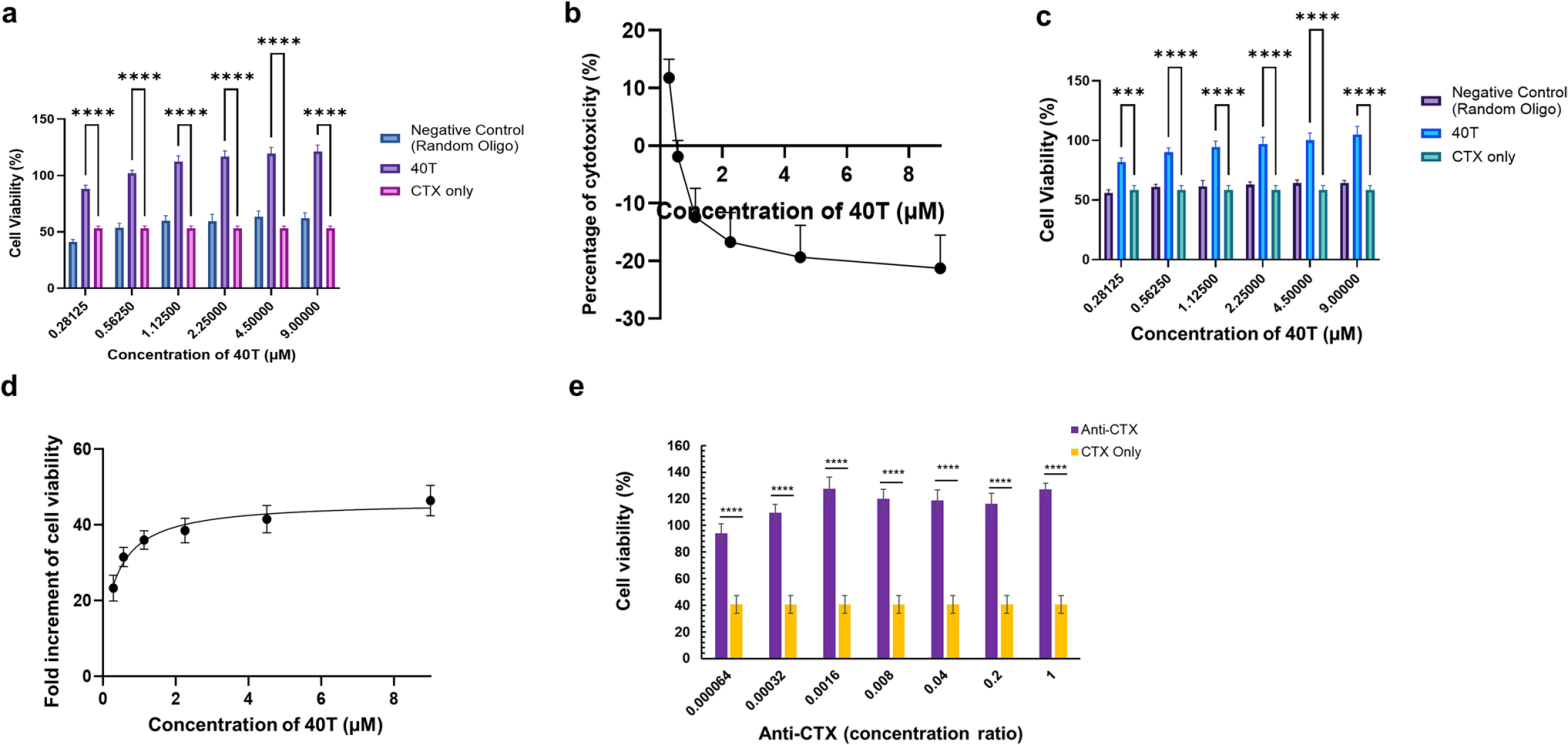
In vitro neutralisation and experimental post-envenomed assay of 40T. **a** Percentage cell viability of in vitro neutralisation assay of 40T. Increasing concentrations of 40T (0.28, 0.56, 1.13, 4.5, and 9 µM) were incubated with CTX for 1 h for complex formation before treatment on HaCaT cells. The cell viability was measured by CCK-8 assay after 24 h of treatment. A negative control of random oligonucleotide of similar length was included in the assay. **b** Percentage of CTX-induced cytotoxicity after in vitro neutralisation of different concentrations of 40T (0.28, 0.56, 1.13, 4.5 and 9 µM). The data was fitted into a non-linear regression model using GraphPad Prism 9. The EC_50_ of 40T was 0.12 µM. **c** Percentage cell viability of aptamer-mediated rescue assay in experimentally post-envenomed HaCaT cells. Increasing concentrations of 40T (0.28, 0.56, 1.13, 4.5, and 9 µM) were treated on CTX post-envenomed cells, and cell viability was measured by CCK-8 assay. **d** Fold increment of cell viability in CTX post-envenomed cells treated with increasing concentrations of 40T. The EC_50_ of 40T was 0.47 µM. **e** Cell viability (%) of experimental post-envenomed HaCaT cells. The HaCaT cells were experimentally envenomed with 9 μM CTX and then treated with anti-CTX scFv with concentration ratios of 0.000064 to 1 for 24 h. All data were expressed as the mean ± S.E.M. of biological and technical replicates (*n* = 3). All data were analysed using two-way ANOVA. ****p ≤ 0.0001; ***p ≤ 0.001; **p ≤ 0.01 and *p ≤ 0.05

To assess the neutralisation potency and efficacy of 40T, in vitro neutralisation and experimental post-envenomed rescue assays were performed. In the neutralisation assay, a concentration-dependent increase in cell viability was observed (Fig. 3a) with increasing concentrations (0.28–9 µM) of 40T. 40T had an EC_50_ of 0.12 µM, which is equivalent to 1.45 µg/mL (Fig. 3b).

On the other hand, in the experimental post-envenomed rescue assay, HaCaT cells were experimentally treated with 9 µM CTX for 6 h to mimic real envenomation conditions. Under the same treatment concentrations (0.28–9 µM), 40T significantly rescued the survival of CTX-envenomed cells (Fig. 3c), with an EC_50_ of 0.47 µM, equivalent to 5.69 µg/mL (Fig. 3d**)**. These findings suggested that 40T attenuated CTX-induced cytotoxicity and restored cell viability.

In this study, we included a monoclonal antibody, anti-CTX scFv TPL0027_01_F7 (Ahmadi et al. 2020), as a positive control in post-envenomed experiments. This anti-CTX scFv exhibited cell rescuing effects against *N. nigricollis* and *N. mossambica* venom fractions at concentration ranges from 72 to 108 µg/mL (Ahmadi et al. 2020). Our results showed that anti-CTX scFv also displayed significant cell rescuing effects (*p* < 0.0001) with an EC_50_ of 1.89 μg/mL (Fig. 3e). 40T had an in vitro and experimental post-envenomed EC_50_s of 1.45 µg/mL and 5.69 µg/mL, respectively. This indicates that 40T had better in vitro neutralisation potency than anti-CTX scFv.

### Importance of structural binding orientation of 40T to CTX’s functional epitopes in the neutralisation of cytotoxicity

CTX is known to cause cell death via several mechanisms, including membrane-damaging effects, apoptosis, necrosis, and necroptosis. Extensive studies have shown that CTX is able to disrupt membrane integrity via its three-fingered functional loops. It penetrates lipid membranes stepwise, starting with a non-insertion mode by electrostatic anchoring at the upper membrane leaflet. Subsequently, membrane permeabilization occurs when all three loops of CTX deform the membrane monolayer leaflet. When the penetration reaches the isotropic phase (CTX–lipid complex formation), it causes membrane deterioration. Finally, CTX forms oligomers with lipid head groups and induces liposome and/or pore formation (Dubovskii et al. 2014). On the other hand, immunoinformatic analyses and epitope-omics also revealed that the epitope sites of CTX are situated at the functional loops that induce cytotoxicity (Hiu et al. 2023). These studies highlighted the importance of the CTX three loops in compromising membrane integrity, followed by subsequent cell death events.

RING analysis revealed that the active residues of CTX interacting with 40T were Pro9, Phe11, Ala17, Lys19, Lys24, Phe26, Pro31, Arg38, Val43, Lys46, Lys52, and Arg60, with the underlined residues situated at the functional epitopes of CTX (Hiu et al. 2023). The basic Lys residues are responsible for membrane interactions (Dubovskii and Utkin 2014). In addition to Lys, Pro is known to influence CTX membrane activity. The Pro9 residue from loop I is important for maintaining the cis-conformer and preventing the incorporation of CTX into the membrane (Dubovskii et al. 2023). In this context, 40T interacted with Pro9 and presumably ‘locking’ the CTX in its cis-form to prevent its cytotoxic effects. Most of the amino acid residues in CTX involved in the binding with 40T were also Lys residues (33%). As 40T is an ssDNA, it possesses an overall negative charge. Therefore, it promotes binding to the positively charged Lys residue through electrostatic interactions.

The binding of 40T to the functional epitopes hinders and prevents CTX–membrane interactions, which explains the neutralising ability of 40T. Molecular dynamics simulations (MDS) revealed loop I of the CTXs facilitates their insertion into the lipid membrane (Konshina et al. 2017, 2021). It can also cause varying membrane-perturbing activities depending on the CTX isomeric conformers (Dubovskii et al. 2023). Apart from loop I, the involvement of loop II in CTX–membrane interactions has also been widely reported (Dubovskii et al. 2018, 2022; Gorai and Sivaraman 2017; Wu et al. 2022). An all-atom MDS study (Gorai et al. 2016) revealed that loop II of the CTX was the primary structural region, particularly its head and ‘loop’ grooves, which consequently induced pore formation and transmembrane leakage (Wu et al. 2010). All three functional loops are crucial for orienting and facilitating the penetration of CTX into membranes (Dubovskii and Utkin 2024).

When comparing the efficacy and potency of 40T to another tRNA-mimetic anti-CTX aptamer, AptRNA6, which we previously discovered (Hiu et al. 2024), we found that the binding orientation and structure of aptamer–CTX complexes play a crucial role in determining the neutralising and rescuing potency of the anti-CTX aptamer. AptRNA6 exhibited neutralising and rescuing potency in nanomolar (nM) scale (Hiu et al. 2024), while 40T had potency in micromolar scale (µM). AptRNA6 possesses a more complex structure (Hiu et al. 2024), unlike 40T which had a linear structure. As revealed by molecular docking analysis, 40T acquired a ‘sandwich’ configuration with CTX (Fig. 2a) while AptRNA6 appeared to shield CTX entirely (Hiu et al. 2024). Only small region of 40T was involved in the interaction with CTX, as compared to AptRNA6, allowing more CTX residues to interact with cell membranes and other venom components which might cause toxin synergism. This explained the importance of binding orientation of aptamer-CTX in its potency. In spite of differences in the selection approach of anti-CTX aptamer, both AptRNA6 and 40T exhibited potency and efficacy in neutralising CTX-induced cytotoxicity. This suggests that repetitive centrifugation-based SELEX approach can complement the computational selection approach in a more robust and comprehensive discovery of target-specific aptamer candidates. A combination of both approaches not only enhances their strengths, but also complements each other’s limitations.

### Neutralisation potency and efficacy of 40T against *Naja* sp. venoms in experimentally post-envenomed model

To assess the neutralisation potency and efficacy of 40T against *Naja* sp. venom-induced cytotoxicity, we chose venom from three Category 1 medically important venomous species from Southeast Asia, namely, *Naja siamensis*, *Naja sputatrix,* and *Naja sumatrana.*

**Fig. 4:**
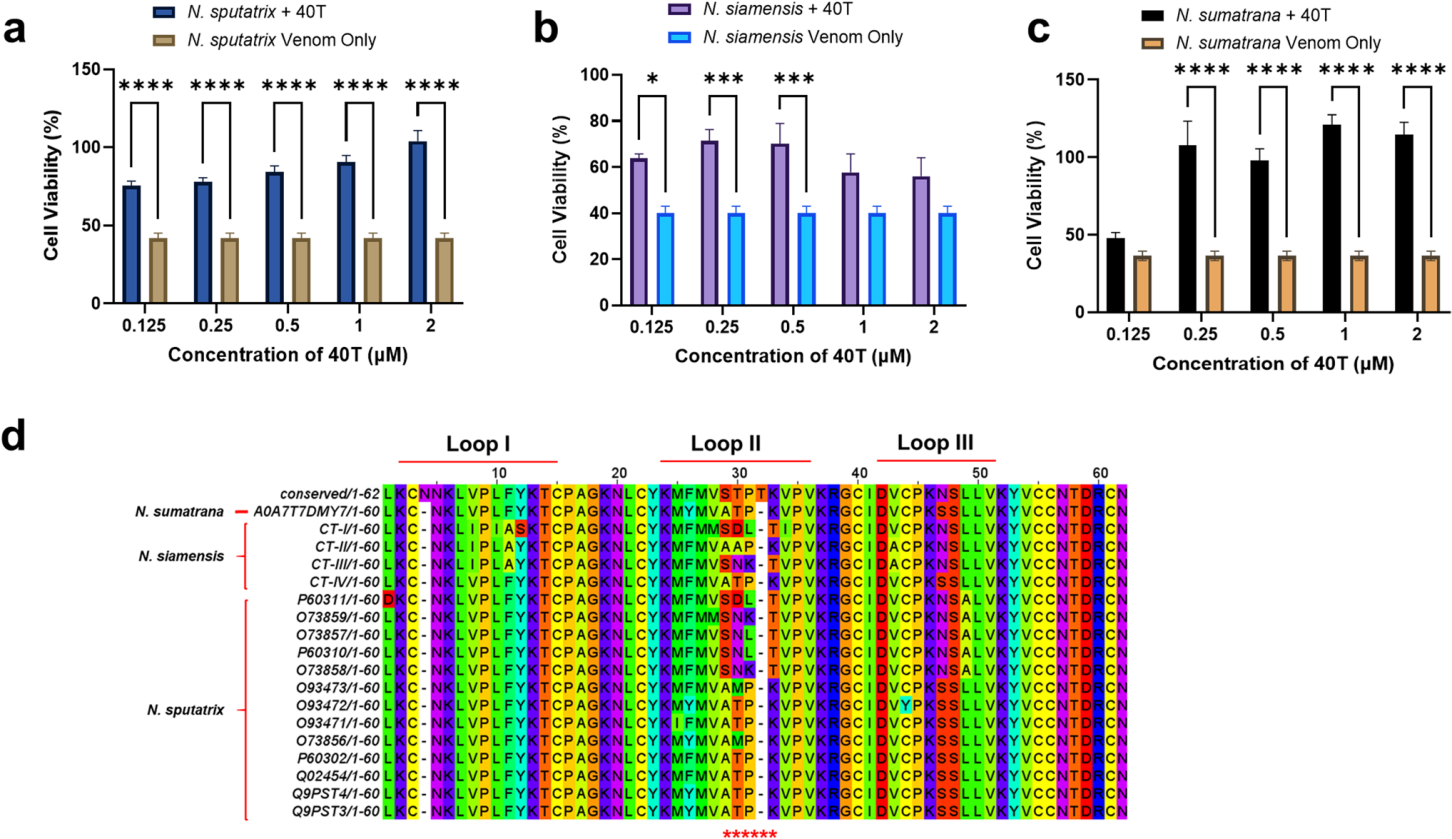
Neutralisation potency and efficacy of 40T in experimentally post-envenomed models with venoms from **a** *N. sputatrix*; **b** *N. siamensis*; **c** *N. sumatrana*. The cell viability (%) of 40T treated experimentally post-envenomed models with the venoms. HaCaT cells were experimentally envenomed with IC_50_ of each venom for 6 h, followed by 40T treatment with 0.125–2 µM for 24 h. All data are shown as mean ± S.E.M. of biological replicates and technical replicates (*n* = 3). ****p ≤ 0.0001, ***p ≤ 0.001, **p ≤ 0.01. **d** Multiple sequence alignment of the conserved CTX sequence (Misuan et al. 2023) and the CTX isoforms from *N. sputatrix*, *N. sumatrana*, and *N. siamensis* venoms. The fasta sequences of CTX isoforms for *N. sputatrix,* and *N. sumatrana* venom were retrieved from UniProt Protein Database (https://www.uniprot.org/), while the sequences of CTX isoforms of *N. siamensis* venom were retrieved from (Ohkura et al. 1988). The asterisk (*) indicated highly variable residues at functional loop II of CTX

CTX compositions of *N. siamensis, N. sputatrix*, and *N. sumatrana* venoms are 21%, 48%, and 44%, respectively (Liu et al. 2017; Tan et al. 2017; Yap et al. 2014). We determined the IC_50_ values of these venoms. Among all the venoms, *N. sumatrana* venom had the lowest IC_50_ (14.21 µg/mL), followed by *N. sputatrix* venom (14.92 µg/mL) and *N. siamensis* venom (28.22 µg/mL). HaCaT cells were experimentally envenomed with the IC_50_ of each venom for 6 h, as it has been reported that dermonecrosis starts to develop beyond the 6 h time point. As shown in Fig. 4a, 40T enhanced the viability of HaCaT cells post-envenomed with *N. sputatrix* venom in a concentration-dependent manner at all concentrations. Nevertheless, the neutralisation potency of 40T was limited to only concentrations of 0.125–0.5 µM and 0.25–2 µM for the *N. siamensis* and *N. sumatrana* experimental models, respectively (Fig. 4b, c). 40T demonstrated notable neutralising effects against these venoms at low concentrations (Fig. 4b, c). The neutralisation efficacy of 40T ranged from 83 to 153% (0.125–2 µM) for *N. sputatrix* venom, 64–82% (0.125–0.5 µM) for *N. siamensis* venom, and 200–240% (0.25–2 µM) for *N. sumatrana* venom. *Naja siamensis* venom has relatively lower abundance of CTX (Liu et al. 2017) to be neutralised by the 40T aptamer.

The variations in the neutralisation potency and efficacy were attributed to the differences in the CTX sequences in each venom. The functional loop II of CTX appeared to be highly variable within different CTX isoforms in the venoms (Fig. 4d). Functional loop II plays a significant role in CTX–membrane interactions, and diversification in loop II among different CTX isoforms contributes to the distinguishable potency and efficacy of 40T in different types of venom-induced cytotoxicity. The results thus highlight the importance of broad specificity in aptamer neutralisation that targets CTX epitopes of different isoforms. Nonetheless, these findings indicated that 40T was able to neutralise venom-induced cytotoxicity even at a lower concentration than the currently available antivenoms. A preclinical evaluation of the cross-neutralisation ability of a bivalent freeze-dried neurotoxic antivenom (FNAV) from Taiwan determined its potency against *N. siamensis* venom to be 230 µg/mL (Liu et al. 2017). On the other hand, the Indonesian antivenom serum anti-Bisa Ular (SABU) exhibited a neutralising potency of 160 µg/mL against CTX isolated from *N. sputatrix* venom (Tan et al. 2017). A comparative cross-neutralisation study of Thai *Naja kaouthia* monovalent antivenom (NkMAV) also demonstrated a degree of neutralisation of 490–902 µg/mL against *N. sumatrana* venoms from Malaysia, Thailand and Indonesia (Tan et al. 2022). Another study using neuro-polyvalent snake antivenom (NPAV) reported a potency of 60 µg/mL against the CTX present in *N. sumatrana* venom (Leong et al. 2015). All these studies have indicated the lower potencies of the currently available antivenoms in neutralising venoms, particularly CTX-induced cytotoxicity.

## Conclusions

This study selected and discovered a 40T from repetitive centrifugation-based SELEX selection and an Illumina amplicon sequencing workflow**.** 40T demonstrated good binding with high specificity for CTX, with a *K*_D_ value of 0.33 µM. The binding of 40T to the functional loop CTX allowed the neutralisation and inhibition of CTX-induced cytotoxicity, with an EC_50_ of 0.12 µM. In the experimental post-envenomed model with CTX, 40T was shown to rescue and restore cell survival in a concentration-dependent manner, with a reported EC_50_ of 0.47 µM. In addition, 40T also exhibited potency and efficacy in experimental post-envenomated models with venom from *N. siamensis*, *N. sputatrix* and *N. sumatrana*, indicating its robust cell-rescuing ability even at low treatment concentrations. These findings suggest that 40T has promising biotherapeutic potential for further engineering in the treatment of dermonecrosis.

## Supplementary Information

Below is the link to the electronic supplementary material.

Supplementary file1 (XLSX 1288 KB)

Supplementary file2 (TIF 1683 KB)

## Data Availability

All data analysed in this study are included in this article. The datasets generated are also available from the corresponding author on reasonable request.
