# Supplementary figures and images for: Targeting cobra venom cytotoxin: a linear 40-mer ssDNA aptamer-based antivenom confers neutralisation potentials against cobra venom-induced cytotoxicity
Source: Arch Toxicol. 2025 Sep 30;100(1):389–403. doi: 10.1007/s00204-025-04211-z (PMC12858506; doi:10.1007/s00204-025-04211-z)

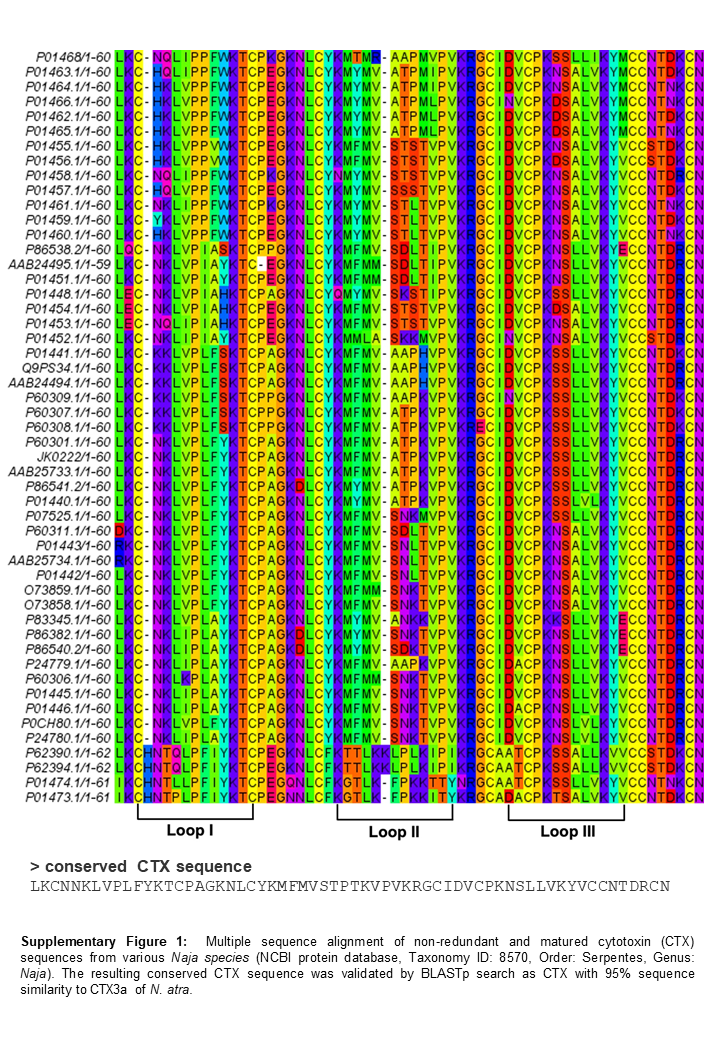

Supplement: Supplementary file 2 — Supplementary file2 (TIF 1683 KB) [file 204_2025_4211_MOESM2_ESM.tif]
